# High-Quality Draft Genome Sequence of *Curtobacterium* sp. Strain Ferrero

**DOI:** 10.1128/genomeA.01378-17

**Published:** 2017-11-30

**Authors:** Ebrahim Osdaghi, Natalia Forero Serna, Stephanie Bolot, Marion Fischer-Le Saux, Marie-Agnès Jacques, Perrine Portier, Sébastien Carrère, Ralf Koebnik

**Affiliations:** a IRD, Cirad, Université de Montpellier, IPME, Montpellier, France; b Department of Plant Protection, College of Agriculture, Shiraz University, Shiraz, Iran; c INRA, Laboratoire des Interactions Plantes Micro-Organismes (LIPM), UMR 441, Castanet-Tolosan, France; d CNRS, Laboratoire des Interactions Plantes Micro-Organismes (LIPM), UMR 2594, Castanet-Tolosan, France; e INRA, Institut de Recherche en Horticulture et Semences (IRHS), UMR 1345 SFR 4207 QUASAV, Beaucouzé, France; f CIRM-CFBP, French Collection for Plant-Associated Bacteria, INRA, IRHS, Angers, France

## Abstract

Here, we present the high-quality draft genome sequence of *Curtobacterium* sp. strain Ferrero, an actinobacterium belonging to a novel species isolated as an environmental contaminant in a bacterial cell culture. The assembled genome of 3,694,888 bp in 49 contigs has a G+C content of 71.6% and contains 3,516 predicted genes.

## GENOME ANNOUNCEMENT

The genus *Curtobacterium* comprises Gram-positive aerobic corynebacteria (family *Microbacteriaceae*, order *Actinomycetales*), including at least 11 well-defined species (1). Although most of the *Curtobacterium* species are soil inhabitants (2, 3), several strains were isolated from plants as epiphytic (4, 5) or endophytic (6–8) bacteria, from dairy processing facilities (9), and from indoor surfaces (10, 11). *Curtobacterium* strains were also identified as potential biocontrol agents to be used against plant-pathogenic fungi (12). *C. flaccumfaciens* appears to have the ability to both colonize plant tissues and infect human organs (13). In 2011, the first well-documented case of *C. flaccumfaciens* human infection was reported in a child with septic arthritis following puncture with a Coxspur hawthorn thorn (13). Furthermore, different pathovars of *C. flaccumfaciens* were described as economically important plant pathogens on annual crops (i.e., dry beans and sugar beet), as well as on ornamental plants (i.e., poinsettia and tulip) (14, 15).

We isolated the yellow-pigmented bacterial strain Ferrero as a bacterial cell culture contaminant at the Institut de Recherche pour le Développement (IRD, Montpellier, France) in 2011. The strain produced Gram-positive domed colonies with entire margins on peptone sucrose agar medium 48 to 72 h postincubation at 28°C. DNA for whole-genome sequencing was extracted using the Wizard genomic DNA purification kit (Promega, Madison WI, USA). Initial phylogenetic analysis of a 1,456-bp fragment of the 16S rRNA gene sequence (16) revealed that strain Ferrero has 99% sequence identity with those of *Curtobacterium* species, while other members of the *Microbacteriaceae* family share only 96% or less sequence identity.

We sequenced strain Ferrero using the Illumina HiSeq 2000 platform (GATC, Germany). The shotgun sequencing yielded 56,895,404 read pairs (36,843,866 100-bp paired-end reads, with an insert size of 250 bp, and 20,051,538 50-bp mate pair reads, with an insert size of 3 kb). A combination of Velvet (17), SOAPdenovo, and SOAPGapCloser (18) yielded 49 contigs ≥500 bp (*N*_50_, 138,897 bp), for a total assembly size of 3,694,888 bp, corresponding to 2,533× coverage. Contigs were annotated with GeneMarkS+ version 4.2 (19), predicting a total of 3,406 protein-coding genes, 54 RNA genes, and 56 pseudogenes.

Average nucleotide identity (ANI) analysis (20) showed that strain Ferrero had only 87% sequence identity with *C. luteum* strain NS184 and *Curtobacterium* sp. strain B8. These ANI values are far below the accepted threshold (95 to 96%) for the definition of prokaryotic species (21). Although the ANI data suggest that strain Ferrero could be defined as a new species, a comprehensive multiphase taxonomic study using the type strains of all existing *Curtobacterium* species is warranted to decipher its precise taxonomic status.

### Accession number(s).

This whole-genome shotgun project has been deposited at DDBJ/EMBL/GenBank under the accession no. NXIA00000000. The version described in this paper is the first version, NXIA01000000. Strain Ferrero has been deposited at CIRM-CFBP, the French Collection for Plant-Associated Bacteria (http://www6.inra.fr/cirm_eng/CFBP-Plant-Associated-Bacteria), with the identifier CFBP 8586.
